# Implementing with intention: advantages, challenges, and tactics to optimally utilize the AXR metric

**DOI:** 10.1017/ash.2024.495

**Published:** 2025-02-12

**Authors:** Alyssa Y. Castillo, Holly M. Frost, Nicole M. Poole, Timothy C. Jenkins, Allan M. Seibert, Edward Stenehjem

**Affiliations:** 1 Department of Medicine, Division of Infectious Diseases, University of Colorado School of Medicine, Aurora, CO, USA; 2 Center for Health Systems Research, Denver Health and Hospital Authority, Denver, CO, USA; 3 Department of Pediatrics, Denver Health and Hospital Authority, Denver, CO, USA; 4 Department of Pediatrics, Division of Infectious Diseases and Epidemiology, University of Colorado School of Medicine, Aurora, CO, USA; 5 Department of Medicine, Division of Infectious Diseases, Denver Health and Hospital Authority, Denver, CO, USA; 6 Division of Infectious Diseases, Intermountain Health, Salt Lake City, UT, USA

## Abstract

Antibiotic utilization for respiratory conditions (AXR) is a new Healthcare Effectiveness Data & Information Set ^®^ (HEDIS^®^) measure designed to complement disease-specific metrics to improve outpatient antibiotic prescribing. Unique challenges include ensuring clinicians understand the metric and establishing appropriate goals within different health systems and service lines. Successful implementation requires awareness of the metric’s limitations and may be enhanced by co-reporting with condition-specific antibiotic use metrics to prioritize local interventions.

## The AXR measure

The National Committee for Quality Assurance (NCQA) maintains the Healthcare Effectiveness Data and Information Set (HEDIS^®^) and develops proprietary performance measures to assess the quality of care and service in the insurance industry.^1^ Over 90% of health plans in the United States utilize HEDIS^®^ measures (including commercial, Medicaid, and Medicare managed plans), and the broad reporting of HEDIS^®^ metrics allows for meaningful comparison of clinician performance within a service line.^1^ Financial incentives exist for payees to optimize HEDIS^®^ metric performance to improve the quality of care delivered to patients.

NCQA has historically reported data on 3 HEDIS^®^ measures related to infections and antimicrobial use: avoidance of antibiotic prescribing for upper respiratory infection (URI) and acute bronchitis/bronchiolitis (AAB), and appropriate testing for group A streptococcus prior to antibiotic dispensing for pharyngitis (CWP).^2^ Higher rates indicate appropriate, non-antibiotic treatment for URI and AAB; similarly, high rates for CWP indicate appropriate testing for group A streptococcus prior to antibiotic prescribing. In 2022, the NCQA introduced antibiotic utilization for respiratory conditions (AXR) as a new HEDIS^®^ measure; this metric was defined as the percentage of episodes in the outpatient setting (including clinic, telephone, emergency visit, observation stay, telephone/telehealth encounter, and e-visit) for patients 3 months of age and older with a diagnosis of a respiratory condition that resulted in an antibiotic dispensing event within 3 days, as depicted below:^3^


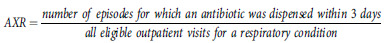




Respiratory conditions included viral or bacterial infections, as well as other illnesses in both the lower and upper respiratory tract.^3^ Patients with a competing diagnosis (defined as a non-respiratory condition for which an antibiotic was indicated) at the presenting encounter or up to 3 days after were excluded.^3^ Patients with comorbid conditions (defined as an immunocompromising condition diagnosed within the 12 months prior to the respiratory-condition encounter) were also excluded.^3^

In contrast to the URI, AAB, and CWP metrics (which measure how often an appropriate decision is made to not prescribe an antibiotic with a target of 100%), AXR measures composite antimicrobial prescribing across a broad range of respiratory conditions.

Given the recent introduction of the AXR metric, implementation experience is limited. Our aim is to highlight the advantages of this metric, identify unique challenges, and propose strategies to optimize its utilization (Table 1).

**Table 1.** Challenges with utilization of the AXR metric and tactics to optimize impact

Challenges	Tactics
**Creating metric meaning for clinicians** • A non-dichotomous metric that includes patients who “sometimes” should receive antibiotics • Need to clearly communicate that metric is benchmarked by service line	• Report and monitor AXR at an organization and service line-level, but report more concrete metrics to clinicians (for example: URI, AAB, CWP) • Report clinician AXR performance over time
**Identifying optimal targets** • Not yet clear what the “ideal” rate is and may vary across different systems, service lines, and geographies • Unclear how to benchmark across systems with variable patient acuity and heterogeneity • Tracking over time will be limited as population case mix varies	• Set initial target at median, with subsequent stepwise decrease over time • Benchmark to similar regional healthcare systems
**Limitations of the metric** • Does not distinguish narrow- vs broad-spectrum antimicrobial use • Does not capture if antibiotic was prescribed at appropriate dose or duration • Does not include antibiotic use in non-respiratory conditions	• Report days of therapy per diagnosis as an additional rate measure • Co-report rates of broad-spectrum antibiotic use or high-risk antimicrobial use
**Incorporating AXR into the HEDIS^®^ metric landscape** • AXR is the 4^th^ antimicrobial stewardship-focused HEDIS^®^ measure • Institutional bandwidth to target all four may be limited	• Target the HEDIS^®^ metric or other clinically impactful, associated stewardship metric with the largest room for improvement, as this will also impact AXR performance
**Assessing Equitable Care** • AXR does not provide granular insight into why antibiotic prescription rates vary between patient groups	• Assess differential trends in AXR performance between groups following stewardship interventions • Engage stakeholders to understand reasons for disparities

Legend: AXR = percentage of episodes for patients 3 months of age and older with a diagnosis of a respiratory condition that resulted in an antibiotic dispensing event, URI = avoidance of antibiotic prescribing for upper respiratory infection, AAB = avoidance of antibiotic prescribing for acute bronchitis/bronchiolitis, CWP = appropriate testing for group A streptococcus prior to antibiotic dispensing for pharyngitis, HEDIS^®^ = Healthcare Effectiveness Data and Information Set

## Advantages

### Encompasses multiple disease-specific metrics

The composite denominator of the AXR metric is comprised of a wide variety of International Classification of Diseases-10 (ICD-10) codes for respiratory diagnoses the clinician specifies during an encounter; this denominator includes diagnoses where antibiotics are justified (Tier 1; for example, bacterial pneumonia), diagnoses where antibiotics are sometimes warranted (Tier 2; for example, sinusitis and acute otitis media), and diagnoses where antibiotics are not warranted (Tier 3; for example, acute uncomplicated bronchitis, cough, and upper respiratory infection).^4^ There are numerous benefits of this broad-sweeping denominator. First, it reduces the need for multiple, disease-specific HEDIS^®^ metrics, as these conditions are all included within the AXR “umbrella” measure. Furthermore, AXR is less susceptible to common cause variation (defined as random variation present in stable healthcare processes), as its denominator is significantly larger than the denominator for disease-specific metrics (like URI and AAB); as a result, it is easier to perceive true special cause variation (defined as unpredictable deviation resulting from a cause that is not an intrinsic part of a process), as would be anticipated following a planned quality improvement initiative.^5^ This also allows for easier identification of variability among clinicians—making the metric more meaningful at the individual clinician level and beneficial to organizations striving for high-reliability care and reduced variability in practice.^6^

### Avoidance of diagnostic drift

It has been well-described that high-prescribing physicians are more likely to diagnose respiratory tract infections that do not require antibiotics as conditions for which antibiotics are sometimes or always necessary (for example, sinusitis)—a phenomenon termed “coding bias.”^7,8^ Such coding bias contributes to “diagnostic drift,” whereby physicians may be more likely to diagnose Tier 1 or 2 conditions than Tier 3 conditions.^8^ In so doing, it is more challenging for stewardship programs to accurately measure rates of inappropriate antibiotic prescribing and provide audit and feedback to high-prescribing clinicians. This “diagnostic drift” also confounds the ability to identify change in antibiotic use over time and assess the true impact of stewardship interventions.^3^ Because AXR is a composite measure of multiple respiratory diagnoses—including Tier 1, 2, and 3 conditions—it is functionally agnostic to these tiers, thus reducing the incentive for coding bias and eliminating the impact of diagnostic drift.

### Broad-range application and benchmarking

The applicability of the AXR measure is broad, as it encompasses all ambulatory care service lines (including primary care, urgent care, and emergency departments for adult and pediatric patients). This is a particular strength of the metric and contrasts with other, condition-specific HEDIS^®^ measures that may be less relevant in certain settings (for example, streptococcal pharyngitis is more common in children than adults, and CWP is thus most insightful in urgent care and pediatric service lines). Furthermore, its broad applicability across all ambulatory care service lines is especially meaningful given that a majority of antibiotics are prescribed in the outpatient setting—and of these antibiotics, the majority are prescribed for respiratory infections.^9,10^ Notably, AXR is the only HEDIS^®^ measure that captures antimicrobial prescribing for sinusitis, which has been established as the top diagnosis associated with antibiotic prescriptions in ambulatory care visits.^10^ As a result, active monitoring and interventions impacting AXR performance can have a large effect on overall outpatient antibiotic prescribing.

Additionally, participating health plans and service lines with access to HEDIS^®^ measure data can readily assess how their clinicians’ performance compares with competitor organizations. Benchmarking is well-established as a successful stewardship tool and is one of the Core Elements of Hospital Antibiotic Stewardship Programs by the Centers for Disease Control and Prevention.^11^ As such, focus on the AXR metric not only allows for high-impact interventions but also enables broad benchmarking to drive ongoing improvement efforts.

## Challenges and strategies

### Ensuring metric understanding and creating meaning for clinicians

#### The challenge

Though the development of AXR as a “utilization metric” is one of its greatest strengths, this also creates challenges in effective interpretation by frontline clinicians. The AXR does not measure the appropriateness of antibiotic prescriptions, as its denominator includes diagnoses for which antibiotics are “sometimes” (Tier 2) or “always” (Tier 1) indicated; that is to say, the target performance will never be 0%. As a result, the absolute rate is less immediately actionable for clinicians, as it does not specify for what diagnoses their prescribing may be above average. The absolute rate is also less informative to clinicians than their performance relative to peers—as the AXR metric can readily identify outlier clinicians whose performance deviates significantly from colleagues in their service line.

In addition, higher-prescribing physicians may seek explanations for why they are an outlier compared to peers. For example, physicians working in an internal medicine clinic may feel that higher rates of antibiotic utilization are justified relative to peers in family medicine clinics. For this reason, it is particularly important to ensure clinicians are aware that the AXR is benchmarked by service line—ie internal medicine clinics are compared only to other internal medicine clinic settings—so that meaningful comparisons with peer facilities can be made. In addition, clinicians often fall victim to the fallacy of believing that their patients are sicker and more likely to require antibiotics than peers’ patients. This misconception is more challenging to address (especially as no risk adjustment is made between clinics within a service line), though emphasizing that the AXR metric excludes patients with certain comorbid conditions (as defined within the HEDIS^®^ Comorbid Conditions Value Set, who may be perceived as higher acuity or higher risk) may alleviate this concern.^12,13^ However, given that the AXR code is proprietary, some organizations have developed internally derived AXR measurements—which, importantly, may not exclude identical (or in some cases, any) comorbidities.

#### Tactics

To optimally utilize the AXR metric while still delivering meaningful and accessible feedback to clinicians, we suggest co-reporting of AXR alongside more concrete, complementary metrics (like URI and AAB). This approach allows clinicians to target their improvement efforts on appropriateness of antibiotics in a specific condition (ie bronchitis) and observe, in parallel, its impact on AXR. If clinicians still find the AXR metric opaque and difficult to interpret, an alternative approach is to monitor AXR at an organization-level while reporting only concrete, disease-specific metrics to clinicians (and providing individual AXR values to requesting clinicians only).

Even for clinicians who lack a nuanced understanding of the AXR measure, providing their AXR trend over time can still serve as powerful feedback. Increases in AXR over time may prompt clinicians to self-reflect and institute changes in their prescribing practices; in contrast, decreases in AXR over time may serve as positive reinforcement for high-performing clinicians who are striving for improvement.

For clinicians who are particularly invested in understanding their antibiotic utilization, providing patient-level data for Tier 3 encounters included in the AXR metric may also be a meaningful intervention. Access to this data enables clinicians to self-audit cases where antibiotics were prescribed to assess the appropriateness of their prescribing and may inform behavior change.

Encouraging organizations to create their own AXR measure with internally derived electronic health record data allows for easier manipulation of the data to co-report complimentary metrics. In addition, it increases the sample size by not limiting the AXR measure to one payor type and makes the measure accessible to a global audience that uses ICD-10 coding.

### Identifying optimal targets

#### The Challenge

Because AXR includes a wide array of respiratory tract diagnoses, it is unclear what the “optimal” or “target” rate should be. In contrast to AAB (which includes exclusively Tier 3 conditions), the AXR rate will always be greater than zero. In addition, it is important to recognize that AXR will vary in response to changes in case mix in the population—and that the “optimal” rate may thus likewise change over time. For example, the AXR would be expected to decrease in the setting of a respiratory viral pandemic, while the AXR would necessarily increase in the setting of high community rates of streptococcal pharyngitis (during which antibiotic prescribing will necessarily rise).^14,15^ In both examples, other metrics would inform interpretation of AXR changes; for example, in the setting of a community outbreak of streptococcal pharyngitis, CWP rates and percent positivity of test results would shed light on the underlying impetus behind changes in AXR.^14^ Consequently, the “optimal” AXR rate will be fluid and prone to change in response to community infection rates, and it is crucial for payors to have timely access to disease-specific data to enable accurate interpretation of AXR trends. This fluidity in response to community infection rates also highlights a challenge with comparison across systems with highly discrepant patient populations (in terms of acuity) or in different geographical locations (where infection outbreaks and case rates may differ).

#### Tactics

One approach is to set the first-year targets at the median rate and subsequently decrease the goal by a pre-determined, incremental amount in subsequent years. This strategy has been adopted by Intermountain Health, which has identified a goal of reducing AXR by 5% per year. It is important to ensure that the median used to inform the target rate is comprised of hospital systems with a similar patient population and case mix to maximize its impact, as highlighted above.

An alternative approach to address this challenge is to not set a target and instead use the metric to identify outlier clinicians, clinic sites, and regions in larger health systems. Notably, providing data on antimicrobial utilization may function as a meaningful intervention even without providing an external goal or target, as outlying high-prescribing clinicians, clinics, and regions may be incentivized to reduce their prescribing based on peer comparison alone, while low-prescribing clinicians may desire to maintain their high-performance.

In addition, AXR trajectory may serve as a harbinger for the durability of stewardship interventions—with rises signaling a potential need to reassess the sustainability of previously-instituted interventions—and serve as an early signal of changes in prescribing culture.

### Limitations of the metric

#### The challenge

While inappropriate prescribing for respiratory conditions is a key area of antibiotic overuse, it is worth noting that there is substantial overprescribing for non-respiratory conditions captured by neither the AXR metric nor the pre-existing URI, AAB, and CWP metrics (including asymptomatic bacteriuria and skin conditions incorrectly diagnosed as cellulitis). Furthermore, AXR captures the dichotomous decision to prescribe an antibiotic but does not reflect whether the right antibiotic, correct dose, or appropriate duration were prescribed. It is well-established that reducing broad-spectrum antimicrobial use and overall duration of therapy are highly effective stewardship interventions—but improvements in either of these areas would not be reflected in the AXR rate, as the overall rate of prescribing would be unchanged.^14^

#### Tactics

Given the important gaps in AXR described above, co-reporting alongside additional modified rate measures is of particular importance. For example, reporting days of therapy per diagnosis (that is, utilizing the AXR metric and multiplying by the number of antibiotic days) would allow for a more comprehensive assessment of overall antibiotic utilization in outpatient settings. This approach has the additional benefit of aligning well with inpatient antibiotic use reporting mechanisms, allowing comparison of antibiotic utilization across systems with integrated outpatient and inpatient systems. Likewise, co-reporting of broad-spectrum antibiotic use rates (for example, reporting the AXR rate with only broad-spectrum or fluoroquinolone use in the numerator) would also allow for more granular understanding of antibiotic selection trends over time, even when overall prescribing rates are stable.^16,17^

### Incorporating AXR into the HEDIS^®^ landscape

#### The challenge

AXR is the fourth antibiotic stewardship-related HEDIS^®^ measure, and it may feel daunting to health plan leaders to identify the human and IT resources to report, monitor, and develop interventions to impact AXR. Likewise, clinicians may feel bandwidth is limited to focus on AXR while maintaining ongoing improvement efforts in URI, AAB, and CWP.

#### Tactics

Though each of the disease-specific HEDIS^®^ metrics provide distinct diagnostic and antibiotic stewardship data, the cases included in each of these measures are also included in the AXR denominator—and as such, performance improvements in any of these three domains will also lead to improvement in AXR. It may therefore be beneficial to focus limited resources on impacting the disease-specific metric with the greatest clinical impact (based on frequency of clinical encounters in each setting) or lowest overall performance, as quality improvement interventions in individual metrics will yield improved AXR rates, as well.

### Assessing equitable care

#### The challenge

Like other HEDIS^®^ metrics, a final key limitation of AXR is its inability to account for differences in antibiotic prescribing rates for respiratory conditions between patient groups (including race, ethnicity, preferred language, and rural vs urban environment) served by a given healthcare organization. That is to say, AXR does not provide information about why a difference in antibiotic prescription rates between patient groups exists—rather, only that one does. A critical evaluation of the differences among patient groups will thus be required to understand why disparities exist and what can be done to eliminate them.

#### Tactics

Since AXR does not yield specific insight into where disparities in care exist, additional data infrastructure is required by health systems to effectively leverage AXR to promote equity in care. To achieve this aim, health systems must prioritize collection and reporting of race, language, and ethnicity data in internal dashboards and reports monitoring antimicrobial use. Monitoring trends in these data alongside AXR following a stewardship intervention or change in care delivery may help clarify if there is differing impact of the change on specific patient groups and guide further evaluation of inequities. Secondly, AXR can be leveraged to engage key patient, community, service line, and organizational stakeholders to understand the potential reasons for variability between patient groups and resolve gaps when inequities are identified.

## Conclusions

The AXR measure is a valuable addition to the reported HEDIS^®^ metrics and is useful in its applicability across all ambulatory service lines, potential to eliminate multiple disease-specific metrics, decreased susceptibility to common cause variation, and de-incentivization of coding bias and subsequent diagnostic drift. However, successful implementation requires intentional planning to ensure clinicians understand and find meaning in the metric, thoughtful identification of optimal targets, awareness of its limitations, and strategic decision-making to guide targets of local stewardship interventions in the realm of respiratory tract infections.
